# Supplementary material for: Public awareness of colorectal cancer symptoms and risk factors, and exploring screening barriers across nine countries: A multi-national cross-sectional study
Source: PLOS Glob Public Health. 2026 Mar 12;6(3):e0005986. doi: 10.1371/journal.pgph.0005986 (PMC12981490; doi:10.1371/journal.pgph.0005986)
Supplement: S1 Table — (DOCX) [file pgph.0005986.s001.docx]

**S1 Table: Awareness regarding colorectal cancer risk factors**

| Characteristic | N | Overall | Working or studying in healthcare | Not working or studying in healthcare | p-value |
| --- | --- | --- | --- | --- | --- |
| Eating less than 5 portions of fruits/vegetables | 13,030 | 4,246 (33%) | 1,866 (38%) | 2,380 (29%) | <0.001** |
| Eating red or processed meat once a day or more | 13,030 | 6,709 (51%) | 2,801 (58%) | 3,908 (48%) | <0.001** |
| Having a diet low in fiber | 13,030 | 7,270 (56%) | 3,257 (67%) | 4,013 (49%) | <0.001** |
| Being overweight (BMI over 25) | 13,030 | 6,641 (51%) | 2,936 (60%) | 3,705 (45%) | <0.001** |
| Being over 70 years old | 13,030 | 7,174 (55%) | 3,434 (71%) | 3,740 (46%) | <0.001** |
| Having a close relative with bowel cancer | 13,030 | 8,376 (64%) | 3,693 (76%) | 4,683 (57%) | <0.001** |
| Having bowel disease (e.g., ulcerative colitis, Crohn's disease) | 13,030 | 9,270 (71%) | 4,042 (83%) | 5,228 (64%) | <0.001** |
| Having diabetes | 13,030 | 4,584 (35%) | 1,991 (41%) | 2,593 (32%) | <0.001** |
| Drinking alcohol | 13,030 | 9,152 (70%) | 3,766 (78%) | 5,386 (66%) | <0.001** |
| Tobacco smoking | 13,030 | 8,581 (66%) | 3,527 (73%) | 5,054 (62%) | <0.001** |

- ** highly significant p-value <0.001
