# Supplementary material for: Public awareness of colorectal cancer symptoms and risk factors, and exploring screening barriers across nine countries: A multi-national cross-sectional study
Source: PLOS Glob Public Health. 2026 Mar 12;6(3):e0005986. doi: 10.1371/journal.pgph.0005986 (PMC12981490; doi:10.1371/journal.pgph.0005986)
Supplement: S1 Text — (DOCX) [file pgph.0005986.s002.docx]

**EARG Collaborators**

| Name | Email | Affiliation |
| --- | --- | --- |
| Osama Mohammad Faisal Sharkas | osama.sharkas500@gmail.com | School of Medicine, The Hashemite University, Zarqa, Jordan |
| Ahmed Amir Samir | ahmedamirsamir58@gmail.com | MBBCH candidate, Faculty of Medicine, Al Azhar University, Nasr City, Cairo, Egypt |
| Nabil Ahmed Albaser | nabilalbaser2020@gmail.com | Faculty of Medicine and Health Sciences, Al-Razi University, Sana’a, Yemen |
| Ali Malik Tiryag | ali.malik@uobasrah.edu.iq | Fundamentals of Nursing department, College of Nursing, University of Basrah, Iraq |
| Warda Rasool | wardarasool@kemu.edu.pk | Faculty of Medicine, King Edward Medical University, Lahore, Pakistan |
| Tajuddeen Adam Wali | tajuddeenadam31@gmail.com | Faculty of Clinical Sciences, Bayero University, Kano, Nigeria |
| MohmmadRjab S. Seder | mohmmadrjabs@gmail.com | Faculty of Medicine and Health Sciences, Palestine Polytechnic University, Hebron, Palestine |
| Moath Salem | Msalm8044@gmail.com | Faculty of Medicine, Damascus University, Damascus, Syria |
| Layan Mahmoud As’ad Audeh | Layanaudeh01@gmail.com | School of Medicine, The Hashemite University, Zarqa, Jordan |
| Ahmad Bashar Saadieh | ahmadbashar910@gmail.com | Faculty of pharmacy, Zarqa University, College of Pharmacy |
| Yasmine H Eisa | 151307@o6u.edu.eg | Department of Public Health and Preventive Medicine, Faculty of Medicine, October 6 University, Giza, Egypt |
| Naglaa Ali | naglaaali11995@gmail.com | Family Medicine Department, Assuit University Hospitals, Egypt |
| Mohammed Sadeg A. Al-Awar | momed.sadeg@gmail.com | Faculty of Medicine and Health Sciences, Amran University and Al-Razi University, Yemen |
| Ghaida'a Ibrahim Ahmed | Ghaidaibrahim1997@gmail.com | Faculty of Medicine and Health Sciences, Al-Razi University, Yemen |
| Olfat Abdullah Mohammed Alfakih | Olfatfakih@gmail.com | Faculty of Medicine and Health Sciences, Al-Razi University, Yemen |
| Hassan Saleh Hassan Al-jibouri | hasan.saleh1206a@conursing.uobaghdad.edu.iq | Higher Health Institute in Al-Muthana, Ministry of Health, Iraq |
| Maher Abdulameer Atiyah | maher.abdulameer@uobasrah.edu.iq | Fundamentals of nursing, College of Nursing, University of Basrah, Iraq |
| Rabbani Mahmoud Daoud | rabbani.daoud@nhs.net | Royal College of Surgeons of Ireland (RCSI), Bahrain |
| Moosa AlHoda | 17221871@rcsi.com | Royal College of Surgeons of Ireland (RCSI), Bahrain |
| Hasan Alauddeen Alomari | 17242479@rcsi.com | Royal College of Surgeons of Ireland (RCSI), Bahrain |
| Farwa Batool | farwab513@gmail.com | Faculty of Medicine, King Edward Medical University, Lahore, Pakistan |
| Zoha Tariq | zohatariq777@gmail.com | Faculty of Medicine, King Edward Medical University, Lahore, Pakistan |
| Syeda Uzma | s.uzma.kcw@gmail.com | Faculty of Medicine, King Edward Medical University, Lahore, Pakistan |
| Issa Kh. Aldababseh | issa.aldababseh@gmail.com | Faculty of Medicine and Health Sciences, Palestine Polytechnic University, Hebron, Palestine |
| Hadi Alabdullah | dr.alabdullah.md@gmail.com | Faculty of Medicine, Hama University, Syria |
| Mahmoud Alwkaa | mahmoudalwkaa@gmail.com | Faculty of Medicine, Alsham Private University, Syria |
