# Supplementary material for: Public awareness of colorectal cancer symptoms and risk factors, and exploring screening barriers across nine countries: A multi-national cross-sectional study
Source: PLOS Glob Public Health. 2026 Mar 12;6(3):e0005986. doi: 10.1371/journal.pgph.0005986 (PMC12981490; doi:10.1371/journal.pgph.0005986)
Supplement: Attachment — Submitted filename: review.docx [file pgph.0005986.s003.docx]

The topic is very interesting. The sample size is very large. The survey covers several countries.

Introduction

In the introduction, it is important to mention similar work that has been done in this area. It is also necessary to focus on the knowledge gap to be filled regarding the phenomenon being studied.

Method

Why the age limit of 18? This needs to be explained.

You explained how the sample size was obtained. However, it is necessary to explain how the individuals were selected. Since the questionnaire is self-administered, there is a possibility of selection bias, such as the exclusion of people who are illiterate.

Results

Online questionnaires are often associated with non-response. Are there any non-respondents, and what is the profile of these non-respondents?

In the results, it is also important to look for barriers specific to each country. If there are differences between countries.

Discussion

We need to discuss selection biases that may be related to non-respondents, education level, internet access, and sampling.
