# Supplementary material for: Public awareness of colorectal cancer symptoms and risk factors, and exploring screening barriers across nine countries: A multi-national cross-sectional study
Source: PLOS Glob Public Health. 2026 Mar 12;6(3):e0005986. doi: 10.1371/journal.pgph.0005986 (PMC12981490; doi:10.1371/journal.pgph.0005986)
Supplement: Attachment — Submitted filename: Response to Reviewers.docx [file pgph.0005986.s005.docx]

# Title: Public Awareness of Colorectal Cancer Symptoms and Risk Factors, and Exploring Screening Barriers Across Nine Countries: A Multi-National Cross-Sectional Study

# Submission ID : PGPH-D-25-03160

# Editor and Reviewers Comments - PLOS Global Public Health Manuscript

We are grateful to the editor and reviewers for their insightful and constructive comments on our manuscript, titled “Public Awareness of Colorectal Cancer Symptoms and Risk Factors, and Exploring Screening Barriers Across Nine Countries: A Multi-National Cross-Sectional Study.” The feedback provided has been invaluable in refining the manuscript, enhancing its clarity, and strengthening the interpretation of our findings.

We have thoroughly addressed all points raised, and a detailed explanation of the revisions is provided below. All changes have been incorporated in the revised manuscript.

## Editor Comments

Thank you for submitting your manuscript to PLOS Global Public Health. After careful consideration, we feel that it has merit but does not fully meet PLOS Global Public Health’s publication criteria as it currently stands. Therefore, we invite you to submit a revised version of the manuscript that addresses the points raised during the review process.

1.The paper presents important findings across countries on an important public health topic. While these countries are predominantly Muslim majority, the authors do not comment on whether dietary or cultural factors sees a higher CRC risk in this population. If this not a consideration, the authors can focus on the reviewer reports and addressing my comment below

**Authors’ Response:** Thank you for this helpful comment. While the participating countries are predominantly Muslim-majority, current evidence does not indicate that Muslim dietary practices independently increase colorectal cancer risk compared with other populations. Known colorectal cancer risk factors are largely related to modifiable lifestyle elements such as high red/processed meat intake, low fiber consumption, physical inactivity, obesity, and smoking, which vary widely across countries regardless of religion or cultural background. As our study was not designed to assess religion-based dietary behavior or cultural dietary adherence, and our questionnaire did not collect data on religious Thank practices or detailed dietary patterns, we believe such interpretation would be speculative. Therefore, we have focused our discussion on established epidemiological and behavioral risk factors rather than religion-specific dietary factors. We have clarified this scope in the manuscript in limitation section.

Lines: 455-461

2. Please see the reviewer reports and address each of them. These will improve the overall readability of the paper. I also suggest that the authors add table 3 as a supplementary table and table 4 should be subsumed into the text and removed. Table 5 mainly presents very similar percentages across rows and can be better summarized in text as well. Table 6 has formatting and presentation issues that the reviewers have also raised. Please revise table 6 in line with standard presentation of unadjusted and adjusted results.

**Authors’ Response** We thank the Editor for the constructive feedback and agree that the suggested restructuring improves clarity and readability. Accordingly, we have:

Moved Table 3 to supplementary material (now Supplementary Table 1). Line: 275

Removed Table 4 and incorporated its key findings into the Results text. Lines: 287-292

For table 5 which is now table 3, we have revised the Results section to provide a concise narrative summary of the main barriers rather than relying solely on the table. The most frequent barriers, such as perceived lack of symptoms, belief in healthy lifestyle protection, absence of family history, fear of diagnosis, fear of the test, embarrassment, and lack of reminders or time, are now clearly summarized in the text. Additionally, we included a figure to visually present these barriers for easier interpretation. We believe this improves clarity and readability of the results. Lines: 295-307

Reformatted Table 6 which is now table 4 present unadjusted and adjusted odds ratios in a clearer, standardized format. Line: 318

We believe these revisions have improved the manuscript presentation.

## Reviewer 1 Comments

The topic is very interesting. The sample size is very large. The survey covers several countries.

Introduction:
In the introduction, it is important to mention similar work that has been done in this area. It is also necessary to focus on the knowledge gap to be filled regarding the phenomenon being studied.

**Authors’ Response:** We thank the reviewer for this suggestion. In the revised manuscript, we have added references to prior studies on CRC awareness and screening barriers in various countries. We also clarified the specific knowledge gap that our study addresses: to our knowledge, no prior research has conducted a multi-country assessment of public awareness of CRC symptoms, risk factors, and screening barriers in LMICs (low- and middle-income countries). These revisions provide a stronger context for our study and clearly justify the need for a cross-national investigation.

Lines: 106-117

Method:
1.Why the age limit of 18? This needs to be explained.

**Authors’ Response:** We thank the reviewer for this comment. The age limit of 18 years and above was chosen because adults are legally able to provide informed consent for participation in research. Additionally, CRC awareness and screening behaviors are most relevant in adult populations, and including individuals under 18 would not align with the study’s objectives. We have added this explanation to the Methods section of the revised manuscript.

Lines: 133-135

2.You explained how the sample size was obtained. However, it is necessary to explain how the individuals were selected. Since the questionnaire is self-administered, there is a possibility of selection bias, such as the exclusion of people who are illiterate.

**Authors’ Response**: We thank the reviewer for this important observation. We have now clarified in the Methods section that participants were recruited using convenience sampling, coordinated by national leaders and their local collaborators in each country. The online self-administered questionnaire was distributed via social media, emails, and direct contacts. We acknowledge that this approach may have excluded individuals without internet access, those with limited digital literacy, or those who are illiterate, potentially introducing selection bias. This limitation has been explicitly discussed in the revised manuscript in both the Methods and Limitations sections.

Lines: 184-190

Results:
1.Online questionnaires are often associated with non-response. Are there any non-respondents, and what is the profile of these non-respondents?

**Authors’ Response**: We thank the reviewer for highlighting this important point. A total of 16,940 individuals were invited to participate in the study. Of these, 3,910 responses were excluded due to ineligibility (e.g., age below 18, incomplete responses) or duplicate entries, leaving a final sample of 13,030 participants. While we did not collect detailed demographic data for individuals who chose not to respond at all, the excluded responses primarily consisted of incomplete surveys or duplicate entries. We have now clarified this in the Methods section and acknowledged it as a potential limitation related to non-response bias in the Limitations section of the revised manuscript.

Lines: 202-204 & 445-447

2.In the results, it is also important to look for barriers specific to each country. If there are differences between countries.

**Authors’ Response:** We thank the reviewer for this insightful comment. While we acknowledge that barriers to colorectal cancer screening may vary between countries, our study was primarily designed to assess overall public awareness and screening barriers across multiple countries. The included countries share similar cultural and lifestyle habits, which supports the rationale for presenting aggregated results. Given the large number of countries and the variability in sample sizes, we focused on the combined analysis to provide a clear multi-country overview.

Lines:304-307

Discussion:
We need to discuss selection biases that may be related to non-respondents, education level, internet access, and sampling.

**Authors’ Response:** We thank the reviewer for raising this important point. We acknowledge that our study may be affected by selection biases. Since the questionnaire was distributed online, participants needed internet access and the ability to complete the survey in English or Arabic, which may have excluded some older adults, rural populations, or individuals with low literacy. Additionally, our use of convenience sampling may have introduced bias toward individuals with higher education levels or greater health awareness. We have now included a discussion of these potential selection biases in the Limitations section of the manuscript.

Lines: 441-447

## Reviewer 2 Comments

Topic: “Public Awareness of Colorectal Cancer Symptoms and Risk Factors, and Exploring Screening Barriers Across Nine Countries: A Multi-Center Cross-Sectional Study.”
--Manuscript Draft Reviewed—19 December 2025
Manuscript Number: PGPH-D-25-03160
Correction to the manuscript:

Please note: The Manuscript Body Formatting Guidelines were not followed. Paragraphs should also be indented. Numeric numbers (for example, 1) should start at Abstract.

**Authors’ Response:** We thank the reviewer for this valuable observation. The manuscript has now been reformatted to comply with journal guidelines. Paragraph formatting, indentation, and numeric consistency have been corrected throughout the manuscript.

Methods: I would rewrite the methods to read: “A cross-sectional study was conducted across multiple centers in nine countries from February 1, 2024, through April 3, 2024, targeting individuals aged 18 years and older. Data were collected via an online questionnaire in collaboration with national leaders and local team members in each country. To examine factors influencing participants’ knowledge, awareness, and screening barriers, logistic regression analyses were employed.”

**Authors’ Response:** We thank the reviewer for this helpful suggestion. We have revised the Abstract Methods section to improve clarity and conciseness. The description of the study design, data collection approach, study period, and analytical methods has now been rewritten accordingly.

Lines: 32-37

Results: I would rewrite the methods to read: “Among the 13,030 participants, awareness of symptoms was highest for "lump in the abdomen" (70%) and lowest for “back pain” (39%). The most recognized risk factor was “history of bowel disease” (71%), and the least recognized was “low fruit/vegetable intake” (33%). The primary barrier to screening was the belief in low personal risk due to the absence of symptoms (63%). Multivariate logistic regression identified working in the healthcare sector as the strongest predictor of high knowledge (aOR = 2.70, 95% CI: 2.50-2.94). Higher knowledge was also associated with males, older age (25-49 years), and university-level education (all p < 0.001).

**Authors’ Response:** We thank the reviewer for this helpful suggestion. We have revised the Abstract Results sections to improve clarity and readability while maintaining accuracy of the study findings.

Lines: 38-46

Line 62 & 63: “Around the world, colorectal cancer (CRC) is the third most common type of cancer and the second leading cause of cancer-related deaths, according to the World Health Organization (WHO, year?).” It is primarily … “Are you talking about CRC?” Please correct to read "CRC."

**Authors’ Response:** We thank the reviewer for this helpful comment. We have clarified the statement by explicitly referring to colorectal cancer (CRC) and added the publication year of the WHO source.

Lines: 61

Line 64: In 2020 alone. This still means in 2020. Please remove the word “alone.”

**Authors’ Response:** Thank you for this helpful suggestion. We have removed the word “alone” to avoid redundancy and improve clarity.

Line: 61

Line 72: “ … face a growing burden.” (Citation needed here).

**Authors’ Response:**We thank the reviewer for highlighting this. We have now included an appropriate reference to support the statement.

Line: 70

Line 74: Should be left aligned.

**Authors’ Response:**We thank the reviewer for pointing this out. The text on line 74 has been adjusted to be left-aligned as requested.

Lines 90-91: “ … countries still lack well-established national screening programs.” Who do you know? Please cite.

**Authors’ Response:**We thank the reviewer for highlighting this. We have now included an appropriate reference to support the statement.

Line: 89

Line 92-93: What are those current guidelines? Please cite?

**Authors’ Response:**We thank the reviewer for this request. We have now specified and cited the guideline in the manuscript. The sentence now reads: “Current guidelines from the American College of Gastroenterology (ACG) recommend colorectal cancer screening in average‑risk individuals between ages 50 and 75 years, with a conditional suggestion to begin screening between ages 45 and 49 years; colonoscopy and fecal immunochemical testing (FIT) are recommended as the primary screening modalities, while other tests (e.g., flexible sigmoidoscopy, multitarget stool DNA, CT colonography) may be considered when these are not possible.

Lines: 89-96

Line 94: Should be left aligned.

**Authors’ Response:**We thank the reviewer for pointing this out. The text on line 94 has been adjusted to be left-aligned as requested.

Line 97: Several barriers hinder individuals from undergoing regular screening for CRC, such as ….. ? Please state these barriers.

**Authors’ Response:**We thank the reviewer for this comment. In response, we have expanded the section to specify commonly reported barriers to CRC screening, supported by relevant literature. These barriers include lack of awareness about CRC and its screening, absence of physician recommendation, fear or anxiety about the procedure or results, embarrassment or cultural stigma, limited access to healthcare services, financial constraints, and misconceptions about screening need.

Lines: 99-104

Line 101: The study aim should be aligned with the current topic. For example, “Public Awareness of Colorectal Cancer Symptoms and Risk Factors, and Exploring Screening Barriers Across Nine Countries (Jordan, Egypt, Iraq, Bahrain, Syria, Pakistan, Palestine, Yemen, and Nigeria).” This is called research alignment. Please rectify.

**Authors’ Response:**We thank the reviewer for this suggestion. The study aim has been revised to explicitly align with the countries studied, clearly reflecting the focus on public awareness of colorectal cancer symptoms and risk factors, as well as on barriers to screening.

Lines: 118-120

Line 111: Is it that you are doing “multi-national” or “multi-centered”? Please be specific.

**Authors’ Response:** We thank the reviewer for this comment. We have revised the title and manuscript text to accurately reflect that this is a multi-national study, conducted across nine countries, rather than a multi-center study.

Lines: 128

Eligibility criteria:
Lines 117-119: The eligibility criteria should be specific as to who is included and who is excluded from the study. This area is not specific. This area is indecisive.

**Authors’ Response:** We thank the reviewer for this comment. The eligibility criteria have been revised to be fully specific, including the inclusion of adults aged 18 years or older residing in the nine study countries who could complete the questionnaire in English or Arabic, and the exclusion of individuals with a personal history of colorectal cancer, those unable to provide informed consent, those who had previously completed the survey, or those who submitted incomplete responses.

Lines: 133-138

Samling:
Lines 123-125: 385 * 9 = 3,465 participants eligible for the study. I assume that these are eligible to participate in the study across nine countries. How many were ineligible? What was the population size? How many had an incomplete survey (started but did not submit)?

**Authors’ Response:** We thank the reviewer for this valuable comment. The sampling process has now been clarified. Convenience sampling was used, and the minimum required sample size of 385 participants per country was calculated using the Raosoft calculator (95% CI, 5% margin of error, 50% response distribution). A total of 16,940 individuals were invited to participate; 3,910 responses were excluded due to ineligibility (age <18 years, residency outside targeted countries, or duplicate entries), resulting in a final analytical sample of 13,030 participants. Countries that achieved ≥385 respondents were included in country-specific analyses, while countries with fewer than 385 respondents were not reported individually; however, their valid responses were retained in the pooled multi-national analysis. These details have been added to the Sampling section for clarity and transparency.

Lines: 141-154

Line 129-130: What was the validated questionnaire? Please state.

**Authors’ Response:** We thank the reviewer for this comment. We have clarified the description of the questionnaire in the Methods section. The awareness component was derived from the Colorectal Cancer Awareness Measure (Colorectal CAM), as used and validated in the study by Al‑Dahshan et al. The barriers component was obtained from a subsequent study by the same authors that developed and translated the barriers items to assess perceived screening barriers in Qatar. The Arabic versions of both components were used without modification, and their prior use in comparable populations supports their validity for our study.

Lines: 157-162

Line 131: You “also” created a self-administered survey.

**Authors’ Response:** We thank the reviewer for this comment. The sentence has been revised to clearly indicate that a self-administered survey was created, without implying additional steps.

Lines: 163

Line: 172-175: You state nine countries but only had approval from Egypt, Yemen, Iraq, and Syria. What happened to the approval from Jordan, Bahrain, Pakistan, Palestine, and Nigeria?

**Authors’ Response:**We thank the reviewer for this comment. Ethical approval was obtained in Egypt, Yemen, Iraq, and Syria. For Jordan, Bahrain, Pakistan, Palestine, and Nigeria, the study used anonymous, self-administered online surveys, which are considered minimal-risk research under local regulations; participants provided electronic informed consent so the study posed minimal risk, and local ethics approval was not required in these countries according to national regulations for anonymous survey-based research. We have clarified this in the Ethics section of the revised manuscript.

Lines: 207-212

Line: 180-182: Categorical data is treated as the dependent variable in most analyses, but not in all cases. Demographic variables are also treated as independent variables; however, the verbiage stated is dependent on the analyses undertaken (logistic, multinomial regression, etc.).

**Authors’ Response:** We thank the reviewer for this comment. The description of categorical variables in the statistical analysis section has been clarified. Categorical variables were summarized with frequencies and percentages, and depending on the analysis, they were treated as either dependent or independent variables. Regression models, including logistic and multinomial regression, were applied appropriately based on the research question.

Lines: 218-221

Lines 200-202: A total of 16,940 participants were invited to participate in the study. However, 3,910 responses were excluded due to ineligible demographics (age restrictions, residency outside targeted countries, or duplicate entries), resulting in a final sample size of 13,030 participants. Some clarification is needed here. Please refer to lines 123-125.

**Authors’ Response:** We thank the reviewer for this comment. We have clarified the description of participant recruitment and exclusions in the Results section to ensure consistency with the Sampling section. The total number of invited participants, the reasons for exclusions, and the final analytical sample are now clearly stated, along with an explanation regarding countries that did not meet the minimum sample threshold.

Lines: 141-144 & 145-147 &202-204

Line 207: I see below in the table, but you mentioned males here 43% (n = 5,567). How many were females (in the same line as the males)? What percentage (57%, n = 7,463)?

**Authors’ Response:** We thank the reviewer for this comment. The Results section has been revised to report both male and female participants along with their corresponding percentages and counts for clarity.

Line: 258-259

Line 220: Caps for Table 2.

**Authors’ Response:** We thank the reviewer for this comment. The title and headers of Table 2 have been updated to follow proper capitalization in accordance with the journal style.

Lines:273

Line 30: Corrections to Table 3 remove the brackets for ease of reading.

**Authors’ Response:** We thank the reviewer for this comment. In accordance with the editor’s request, Table 3 has been removed from the main manuscript and is now presented as Supplementary Table 1.

Line: 275

Line 247: I would recommend that tables stay on separate pages—not hanging.

**Authors’ Response:** We thank the reviewer for this valuable suggestion regarding table presentation. In alignment with the Editor’s guidance, the table previously located at line 247 has been removed from the manuscript to improve clarity and presentation. The relevant information is now incorporated into the Results section narrative to ensure continuity and readability. Additionally, tables in the revised manuscript have been formatted to ensure appropriate placement and readability.

Line 253: (Table 5). Remove brackets.

**Authors’ Response:** We thank the reviewer for this comment. Brackets around the table 5 reference (now table 3) have been removed in the text to improve readability.

Line: 295

Line 292: “Among all the types of cancer, CRC is ranked as the second type that causes mortality.” According to whom? Please state.

**Authors’ Response:** We thank the reviewer for this comment. The sentence regarding colorectal cancer mortality has been updated to include a citation to authoritative data from the World Health Organization indicating that colorectal cancer is the second leading cause of cancer‑related deaths globally.

Line: 330

Line 292-324: Areas should be consistent with spaces.

**Authors’ Response:** We thank the reviewer for this comment. We have reviewed lines 292–324 and corrected spacing inconsistencies to ensure uniform formatting throughout the text.

## Reviewer 3 Comments

Data Availability:

The current data availability statement does not meet PLOS standards. Authors should deposit the anonymized dataset in a public repository (e.g., Figshare, Dryad, or OSF) to ensure full transparency and reproducibility.

**Authors’ Response:** We thank the reviewer for this important comment. In accordance with PLOS data transparency requirements, we have deposited the fully anonymized dataset in a publicly accessible repository, available on the Open Science Framework (OSF) at: [**https://doi.org/10.17605/OSF.IO/5FEHM**](https://doi.org/10.17605/OSF.IO/5FEHM) . The Data Availability Statement in the revised manuscript has been updated accordingly.

Lines: 531-534

Country Selection and Justification: The rationale for selecting these specific nine countries is unclear. The abstract mentions nine countries, but Table 1 shows 13 countries (including Bangladesh, Saudi Arabia, Sudan, and UAE not mentioned in the methods). This discrepancy needs clarification. Please explain:

Why these countries were selected
The inclusion criteria for countries
How the additional four countries were incorporated

**Authors’ Response:** We thank the reviewer for highlighting this important clarification. The study initially targeted nine countries based on geographic representation, feasibility of recruitment, and availability of research collaborators. During survey dissemination, additional eligible responses were received from Bangladesh, Saudi Arabia, Sudan, and UAE. These responses were retained in the overall pooled dataset; however, these countries were not analyzed individually due to smaller sample sizes. We have now revised the Methods section and added a note to Table 1 to clearly explain the rationale, inclusion criteria, and incorp oration of the additional four countries.

Lines: 145-154 & 252-254


Sample Size Calculation: While you mention needing 385 responses per country using Raosoft calculator, several countries fall below this threshold (e.g., UAE=200, Sudan=368, Bangladesh=256). Please address:

How this affects the representativeness of findings
Whether country-specific analyses are adequately powered
Consider stratified analysis or acknowledging this as a limitation

**Authors’ Response:** We thank the reviewer for this important observation. The minimum required sample size per country was 385 participants based on the Raosoft calculator. While most countries achieved this number, UAE, Sudan, and Bangladesh did not reach the required threshold. We acknowledge that this limits the statistical power and representativeness of country-specific analyses for these countries.

To address this, we revised the Methods section to clarify that:
• data from these countries were included in the pooled multinational analysis
• however, they were not interpreted as nationally representative
• country-specific inferential conclusions were restricted to adequately powered countries

These clarifications have now been incorporated into the revised manuscript.

Lines: 140-141 & 145-154


Knowledge Scoring Methodology: The 70% cut-off for "high knowledge" appears arbitrary. Please provide:

Justification for this specific threshold
Whether this has been validated in previous studies
Sensitivity analysis with different cut-offs

**Authors’ Response:** We thank the reviewer for this comment. The Colorectal CAM instrument we used is a validated and reliable tool for measuring awareness, as shown in Power et al. (2011), but it does not prescribe a specific score cut-off for “high knowledge.” In the absence of a defined threshold, we adopted ≥70% as a pragmatic benchmark to represent attainment of a substantial majority of correct knowledge items, consistent with conventions in public health knowledge assessment. We also examined alternative cut-offs (60% and 80%) and found that the overall patterns and conclusions remained consistent. We have clarified this rationale in the Methods section.

Lines: 222-229

Ethical Approval Inconsistency: Ethics approval was obtained from only 4 countries (Egypt, Yemen, Iraq, Syria) out of 9-13 countries. Please clarify:

Why ethics approval wasn't obtained from all participating countries
Whether institutional review was conducted for countries not listed
How ethical standards were maintained across all sites

**Authors’ Response:** Thank you for raising this important point. Ethical approval was formally obtained from the Institutional Review Boards in Egypt, Yemen, Iraq, and Syria, which provided primary oversight for the multi-national study. The study involved an anonymous, voluntary online questionnaire with no collection of identifiable data and no clinical intervention; therefore, it was considered minimal-risk research.

In the remaining participating countries, local regulations do not require separate IRB/ethical approval for anonymous, non-interventional public awareness surveys and allow reliance on approval from a lead ethical body in multi-national studies. Nevertheless, all procedures adhered to the Declaration of Helsinki, participants provided informed consent electronically, participation was voluntary, and confidentiality was strictly maintained.

We have clarified this in the revised manuscript.

Lines: 207-212


Statistical Methods:

The chi-square tests comparing healthcare vs. non-healthcare workers are appropriate, but consider adding effect sizes (e.g., Cramér's V) to complement p-values

**Authors’ Response:** We thank the reviewer for this suggestion. While Table 4 has been removed from the main manuscript per the editor’s guidance, we have summarized the comparisons between healthcare and non-healthcare participants in the text, including effect sizes (Cramér’s V) to indicate the magnitude of associations.

Lines: 288

For the logistic regression, consider reporting model fit statistics (e.g., AIC, Hosmer-Lemeshow test) Consider testing for multicollinearity among predictors in the multivariate model

**Authors’ Response:** We thank the reviewer for the insightful suggestions regarding the logistic regression analysis. Logistic regression table has been reformatted to clearly present univariate (crude) and multivariate (adjusted) odds ratios with 95% confidence intervals and p-values, with reference categories clearly indicated.

Although the original analyst is no longer available to rerun additional diagnostics, the logistic regression model was assessed at the time of analysis for goodness-of-fit and multicollinearity, and all assumptions were met according to standard procedures. This has now been clarified in both the Methods section and in the Notes of logistic regression table.

Lines: 234-238


Discussion of Country-Specific Variations: Given the cultural and healthcare system differences across countries, the lack of country-stratified analysis is a limitation. Consider: Adding supplementary tables showing country-specific knowledge levels
Discussing how healthcare infrastructure differences might explain variations
Acknowledging regional heterogeneity more explicitly

**Authors’ Response:** We thank the reviewer for this suggestion. While country-specific variations are possible, the nine countries included in this study share broadly similar cultural practices, dietary habits, and healthcare approaches, which supports a pooled analysis. Nevertheless, we acknowledge that regional differences could exist, and we have clarified in the Discussion that this may limit the generalizability of results to individual countries.

Lines: 455-456


Gender Findings: The finding that males have higher knowledge contradicts some cited studies. The discussion mentions this briefly (line 373-374) but doesn't explore potential explanations. Consider discussing: Cultural factors affecting health literacy by gender
Selection bias in your sample (online recruitment may favor certain demographics)
How this varies by country

**Authors’ Response:** We thank the reviewer for this insightful comment. We acknowledge that our finding of higher knowledge levels among males contrasts with some previous studies. Potential explanations include cultural factors that may influence health literacy and information-seeking behaviors differently by gender. Additionally, the online recruitment method may have introduced selection bias, favoring males who are more active on social media or more likely to participate in online surveys. While country-specific gender differences were not analyzed, the pooled sample included countries with broadly similar cultural and healthcare contexts, which we have now clarified in the Discussion. These considerations have been added to provide a more nuanced interpretation of the gender findings.

Lines: 411-418


Age Categorization: The age grouping (<25, 25-49, ≥50) seems inconsistent with CRC screening guidelines (typically 45/50-75 years). Consider: Regrouping to align with screening-eligible populations, Separate analysis for screening-eligible age groups, Justification for current categories.

**Authors’ Response:** We thank the reviewer for this comment. At the time of analysis, colorectal cancer (CRC) screening guidelines recommended screening for individuals aged 50–75 years; therefore, our age categories and analyses were based on this recommendation. The age grouping (<25, 25–49, ≥50) was chosen to capture broad demographic differences in knowledge across younger adults, middle-aged adults, and those approaching or within the screening-eligible population. This approach allowed us to examine trends in awareness across the general adult population while still including the previously recommended screening-eligible group. We acknowledge that current guidelines now recommend starting screening at age 45, and this has been clarified in the Methods. The ≥50 age group in our study corresponds to the lower end of the previously recommended screening-eligible population, and the findings remain informative for public health awareness and educational interventions.

Lines: 238-242


Abstract: Line 46-47 states working in healthcare is the "strongest predictor" (aOR=2.70) but doesn't compare this to the reference category clearly. Specify that this is compared to non-healthcare workers.

**Authors’ Response:** We appreciate the reviewer’s observation. To improve clarity, we have revised the abstract to explicitly note the reference group. The text now states: “Multivariate logistic regression identified working in the healthcare sector as the strongest predictor of high knowledge (aOR = 2.70, 95% CI: 2.50–2.94) relative to non-healthcare participants”

Line: 43-45

Introduction:

Line 67: "More than 80%" - please provide the specific reference

**Authors’ Response:** Thank you for this helpful comment. We agree that the statement requires a supporting reference. We have now added an appropriate citation

Line: 75

Line 92-94: Update screening age recommendations as recent guidelines now suggest starting at 45 years for average-risk individuals

**Authors’ Response:** Thank you for this important comment. We agree that screening age recommendations have evolved. We have now updated the manuscript to reflect the most recent international guidelines, which recommend initiating colorectal cancer screening at 45 years for average-risk individuals. At the time of our study and analysis, the commonly adopted threshold was 50 years; however, we acknowledge this change and have clarified it in the revised text.

Lines: 95-96


Methods:

Line 123-125: "Convenience sampling" - acknowledge this as a limitation affecting generalizability

**Authors’ Response:** We thank the reviewer for this important observation. We agree that the use of convenience sampling may introduce selection bias and limits the generalizability of our findings. We have now explicitly acknowledged this as a limitation in the revised manuscript and clarified its potential impact on interpretation of results.

Lines: 445-447

Line 131-132: Specify which modifications (if any) were made to the validated questionnaire

**Authors’ Response:** Thank you for this helpful comment. The questionnaire used in our study was fully adopted from previously validated instruments developed in Qatar (Al-Dahshan et al., 2020; Al-Dahshan et al., 2021). We confirm that **no modifications were made** to the wording, structure, content, or response options of the questionnaire. The tool had already undergone cultural adaptation and validation in both Arabic and English, which made it suitable for our study population. This clarification has now been added to the Methods section of the revised manuscript

Lines: 157-162

Line 165-166: Clarify how duplicate entries were identified

**Authors’ Response:** Thank you for this helpful comment. Duplicate entries were prevented through an initial screening question at the start of the survey asking whether the participant had previously completed the questionnaire. If “Yes” was selected, the survey automatically terminated, and the response was excluded. This clarification has now been added to the Methods section.

Lines: 195-198


Results:

Table 1: The p-value for age (0.027) is significant at p<0.05 but text states "only age showed no significant association" - this appears contradictory

**Authors’ Response:** Thank you for pointing out this inconsistency. The p-value for age (0.027) indicates a statistically significant association with working in the healthcare sector. The text stating that “only age showed no significant association” was incorrect. We have corrected this in the Results section to accurately reflect the significant association of age, consistent with Table 1.

Lines: 261

Line 243-244: "31% demonstrated high knowledge" - provide breakdown by country as this likely varies substantially

**Authors’ Response:** We thank the reviewer for this comment. We did not perform country-specific analyses because the included countries share broadly similar cultural practices, dietary habits, and healthcare approaches, which supports a pooled analysis. While we acknowledge that knowledge levels may vary slightly.


Discussion: 284-286

Line 292: "CRC is ranked as the second type that causes mortality" - globally, yes, but this may differ by country/region

**Authors’ Response:** Thank you for this important comment. We have clarified in the manuscript that the statement refers to **global colorectal cancer mortality**, and we note that rankings may vary by country or region.

Line: 329-330

Line 337-342: The comparison to studies with different populations is appropriate, but acknowledge how this limits direct comparability

**Authors’ Response:** We thank the reviewer for this comment. We have clarified in the manuscript that differences in study populations—such as age restrictions, recruitment from specific educational or occupational groups, or single-country settings—limit the direct comparability of previous studies to our multi-country general adult sample. The revised text now explicitly acknowledges this limitation

Lines: 378-380

Lines 362-371: The addition about Iraq, Turkey, UAE, and Norway is valuable but feels somewhat disconnected. Consider integrating more smoothly or expanding

**Authors’ Response:** We thank the reviewer for this comment. We have revised the paragraph to improve flow and clarity by logically grouping the discussion of barriers and successful programs across countries. The text now smoothly integrates examples from Iraq, Turkey, UAE, Norway, and the UK, and links them to practical recommendations such as implementing national screening programs and introducing FIT as a non-invasive alternative

Lines: 400-409

Line 373-386: Good comparison with literature, but consider organizing by theme rather than country

**Authors’ Response:** We thank the reviewer for this comment. We have reorganized the discussion by theme (gender, age, education, and occupation) rather than by country, using only the information from the original studies cited. This improves readability and facilitates clearer comparison with the literature.

Lines: 419-429

Limitations Section (Lines 396-402):

Excellent acknowledgment of online survey bias
Add: convenience sampling, country selection, lack of country-specific analysis
Add: potential recall bias in self-reported knowledge
Add: cross-sectional design preventing causal inference

**Authors’ Response:** We thank the reviewer for highlighting these important considerations. We have revised the Limitations section to explicitly acknowledge online survey bias, convenience sampling, country selection, lack of country-specific analysis, potential recall bias in self-reported knowledge, and the inability to infer causality due to the cross-sectional study design.

Lines: 440-447


Language and Presentation:

Line 68: "changes in lifestyle and dietary habits" - be more specific

**Authors’ Response:** We thank the reviewer for this comment. We have revised the text to specify the lifestyle and dietary factors relevant to colorectal cancer risk, including physical inactivity, tobacco and alcohol use, high red/processed meat consumption, and low fruit and vegetable intake

Lines: 66-68

Line 337: "It is worth mentioning" - remove informal phrase

**Authors’ Response:** We thank the reviewer for this comment. The informal phrase “It is worth mentioning” has been removed, and the sentence has been revised to improve clarity and formality.

Line: 374-375

Consider consistent terminology: "bowel cancer" vs "colorectal cancer"

**Authors’ Response:** We thank the reviewer for this comment. We have standardized terminology throughout the manuscript, using “colorectal cancer (CRC)” consistently instead of “bowel cancer” to improve clarity and consistency.


Figures:

Figures 1-3 are clear and well-presented
Consider adding error bars or confidence intervals
In Figure 3, consider reordering from highest to lowest percentage for easier interpretation

**Authors’ Response:** We thank the reviewer for this helpful suggestion. Figures 1–3 present descriptive proportions of awareness items to facilitate visual comparison across symptoms and barriers. Confidence intervals were not included because these figures are intended for descriptive visualization rather than inferential comparison, and the large overall sample size minimizes random variation. To improve clarity, we have reordered Figure 3 from highest to lowest percentage as suggested.


References:

Reference 2 appears incorrect - cites an antiemetics guideline rather than CRC epidemiology

**Authors’ Response:** We thank the reviewer for pointing this out. You are correct that reference 2 (Hesketh et al., 2020) is not relevant to colorectal cancer epidemiology. This was included in error, and we have replaced it with the appropriate reference on CRC epidemiology.

Line: 68 & 70

Ensure all references are current (several from 2011-2017 may have updates)

**Authors’ Response:** We thank the reviewer for this comment. While some references are from 2011–2017, we believe they remain important because they provide foundational evidence and context for the study of colorectal cancer awareness and screening practices. We have also included recent references to ensure the manuscript reflects current guidelines and developments.

## Reviewer 4 Comments

I applaud the authors for such a great and important work.
1. The first three sentences of the introduction need references.

**Authors’ Response:** We thank the reviewer for the positive feedback and valuable suggestion. We agree that the first three introductory statements require appropriate citations. We have now added relevant references to support these statements in the revised manuscript

Lines: 61

2. One limitation mentioned is younger respondents answering screening limitation questions; authors should conduct subgroup analysis restricted to screening-age individuals.

**Authors’ Response:** We sincerely thank the reviewer for this valuable suggestion. We fully acknowledge the importance of examining outcomes specifically among individuals within the screening-eligible age group. However, subgroup analysis was not included in the original analytical plan, and the study was not specifically powered to detect statistically meaningful associations within the screening-eligible age population alone.

In addition, the primary objective of this study was to assess general awareness and knowledge of colorectal cancer across the adult population rather than solely screening uptake among older individuals. Including younger adults was intentional, as this group represents an important target population for early education, especially given the increasing global focus on earlier screening and rising CRC incidence in younger adults.

Nevertheless, we agree that the inclusion of a broader age range may influence interpretation of screening-related findings. To address this important point, we have strengthened the manuscript by clearly acknowledging this as a methodological limitation and clarifying that screening behavior among screening-age individuals may differ from the general adult sample. This has now been explicitly stated in the limitations section.

Lines: 447-455
